# Isolation and Characterization of 46 Novel Polymorphic EST-Simple Sequence Repeats (SSR) Markers in Two Sinipercine Fishes (*Siniperca*) and Cross-Species Amplification

**DOI:** 10.3390/ijms13089534

**Published:** 2012-07-30

**Authors:** Chunmei Qu, Xufang Liang, Wei Huang, Liang Cao

**Affiliations:** College of Fisheries, Huazhong Agricultural University, Wuhan 430070, China; E-Mails: linghanliuxiang@163.com (C.Q.); huangwei880924@163.com (W.H.); caoliang0205@163.com (L.C.)

**Keywords:** *Siniperca chuatsi*, *Siniperca scherzeri*, EST-SSRs, transcriptome, cross-species amplification

## Abstract

With the development of next generation sequencing technologies, transcriptome level sequence collections are emerging as prominent resources for the discovery of gene-based molecular markers. In this study, we described the isolation and characterization of 46 novel polymorphic microsatellite loci for *Siniperca chuatsi* and *Siniperca scherzeri* from the transcriptome of their F_1_ interspecies hybrids. Forty-three of these loci were polymorphic in *S. chuatsi*, and 20 were polymorphic in *S. scherzeri*. In *S. chuatsi*, the number of alleles per locus ranged from 2 to 8, and the observed and expected heterozygosities varied from 0.13 to 1.00 and from 0.33 to 0.85, respectively. In *S. scherzeri*, the number of alleles per locus ranged from 3 to 9, and the observed and expected heterozygosities varied from 0.19 to 1.00 and from 0.28 to 0.88, respectively. We also evaluated the cross-amplification of 46 polymorphic loci in four species of sinipercine fishes: *Siniperca kneri*, *Siniperca undulata*, *Siniperca obscura*, and *Coreoperca whiteheadi*. The interspecies cross-amplification rate was very high, totaling 94% of the 184 locus/taxon combinations tested. These markers will be a valuable resource for population genetic studies in sinipercine fishes.

## 1. Introduction

Mandarin fish (*Siniperca chuatsi*), an economically important species in China, has a relatively high market value, and is wide cultured throughout the country [1,2]. It has a fast growth rate, but is susceptible to diseases. Compared with *S. chuatsi*, Golden mandarin fish (*Siniperca scherzeri*) has a great disease resistance, but grows slowly. Recently, outbreaks of diseases caused by parasites, bacteria and viruses have caused severe economic losses to the aquaculture industry [3]. In addition, because of overfishing, drought and especially water pollution, the wild stock of *S. chuatsi* is declining [4]. Therefore, breeding a disease-resistant and faster growing strain and preserving fish germplasm are becoming urgent aims in China.

Microsatellites or simple sequence repeats (SSRs) have become a useful tool to assess genetic diversity and develop molecular breeding techniques in fish due to their co-dominance, ubiquitous distribution within genomes, high reproducibility, and transferability across species [5,6]. However, the development of microsatellite markers has been limited by the labor and time required to construct, enrich, and sequence genomic libraries [7]. Fortunately, with the advent of next generation sequencing technologies, transcriptome sequencing is emerging as a rapid and efficient means for gene discovery and genetic marker development. Since EST-SSRs derived from transcriptome exist in the transcribed region of the genome, they can lead to the development of gene-based maps which help to identify candidate function genes and increase the efficiency of marker-assisted selection (MAS) [8]. Furthermore, EST-SSRs show a higher level of transferability to closely related species than non-EST-SSRs [9].

Although a few microsatellite markers were developed for *S. chuatsi* [10–14] and *S. scherzeri* [15], the number of available SSRs is grossly inadequate for genetic and mapping studies. Here, we describe the isolation and characterization of 46 novel polymorphic microsatellite loci for the *S. chuatsi* and *S. scherzeri*. We also test the transferability of these markers in other four species of sinipercine fishes: *Siniperca kneri*, *Siniperca undulata*, *Siniperca obscura*, and *Coreoperca whiteheadi*.

## 2. Results and Discussion

As shown in Table 1, a total of 46 polymorphic EST-SSR markers were newly developed. Forty-three of these loci were polymorphic in *S. chuatsi*, and 20 were polymorphic in *S. scherzeri*. Concerning *S. chuatsi*, the number of alleles per locus ranged from 2 to 8, with an average of 4.3 alleles per locus. The observed (*H_O_*) and expected heterozygosities (*H_E_*) ranged from 0.13 to 1.00 (average of 0.55) and from 0.33 to 0.85 (average of 0.63), respectively. In *S. scherzeri*, the number of alleles per locus ranged from 3 to 9, with an average of 5.5 alleles per locus. The observed (*H_O_*) and expected heterozygosities (*H_E_*) ranged from 0.19 to 1.00 (average of 0.74) and from 0.28 to 0.88 (average of 0.72), respectively. Five loci (Sin134 in *S. chuatsi*, Sin118, Sin122, Sin158 and Sin159 in *S. scherzeri*) showed significant deviation from the Hardy-Weinberg equilibrium (HWE) after Bonferroni correction (adjusted *p*-value = 0.0012 for *S. chuatsi* and 0.0026 for *S. scherzeri*), which may be due to the small sample size (*n* = 32) or the excess of heterozygotes. Another possible explanation for the departure from HWE is the dramatic contemporary decline in spawning populations, and consequent non-random mating and genetic bottlenecks [14]. No evidence for allelic dropout was found in these loci. No significant linkage disequilibrium (LD) was detected across all loci following Bonferroni correction (adjusted *p*-value = 0.0001 for *S. chuatsi* and 0.0003 for *S. scherzeri*).

Overall, a high level of cross-species amplification was observed across the four species (Table 2). Forty-five of 46 polymorphic loci (97.8%) were amplified successfully in *S. undulate* and *S. obscura*, 44 (95.7%) in *S. kneri*, and 39 (84.8%) in *C. whiteheadi*. These results were expected because of the taxonomical relationships of the families [16]. *S. kneri*, *S. undulata*, *S. obscura* are closely related to *S. chuatsi* and *S. scherzeri*, and all species belong to *Siniperca*, whereas *C. whiteheadi* is from *Coreoperca* which is sister genera to *Siniperca*. As transcriptome sequences are typically conserved relative to nontranscribed regions, SSRs residing in transcriptome sequences typically benefit from higher amplification rates and higher levels of cross-species transferability [17,18]. The high level of cross-species amplification tested here indicated not only the potential utility of transcriptome sequences for the identification and characterization of large numbers of gene-based SSR loci across species for which limited marker resources were available, but also the potential usefulness of the developed markers for a broader range of evolutionary, conservation and management studies in sinipercine fishes.

## 3. Experimental Section

*De novo* transcriptome sequencing of F_1_ hybrids between *S. chuatsi* (♀) and *S. scherzeri* (♂) was performed and a total of 118,218 unigenes were identified. The processes of library preparation for transcriptome analysis and sequence assembly were as described in [19]. This unigene set was used for mining EST-SSR markers using the default parameters of the BatchPrimer3 v1.0 software [20]. In this study, a subset of 62 EST-SSR markers was screened on 32 *S. chuatsi* (Chibi, Hubei Province, China) and 32 *S. scherzeri* (Fengcheng, Liaoning Province, China), respectively. The primers for these SSR loci were designed using NCBI/Primer-BLAST [21].

Total genomic DNA was extracted from fin clips using the TIANamp Genomic DNA Kit (Tiangen) following the manufacturer’s instructions. Polymerase chain reaction (PCR) conditions were optimized for each pair of primers. PCRs were performed in 25 μL reaction volumes containing 2.5 μL of 10× PCR buffer, 1.0–3.0 mM MgCl_2_, 50 μM dNTPs, 0.4 μM of each primer, 1 U *Taq* polymerase (Takara) and 50 ng genomic DNA. PCR conditions were as follows: initial denaturation at 94 °C for 3 min followed by 35 cycles at 94 °C for 30 s, the optimized annealing temperature (Table 1) for 30 s, 72 °C for 30 s, and then a final extension step at 72 °C for 10 min. PCR products were separated on a 8% non-denaturing polyacrylamide gel electrophoresis and visualized by silver staining. A denatured pBR322 DNA/MspI molecular weight marker (Tiangen) was used as a size standard to identify alleles.

The number of alleles (*Na*), the observed (*H_O_*) and expected heterozygosities (*H_E_*) were estimated using POPGENE version 1.32 [22]. The polymorphic information content (*PIC*) was calculated using the formula:

$$PIC=1-(\sum_{i=1}^{n}q_{i}^{2})-(\sum_{i=1}^{n-1}\sum_{j=i+1}^{n}2q_{i}^{2}q_{j}^{2})$$

where *n* is the number of alleles, and *q_i_*, *q_j_* is the *i*th and *j*th allele frequency, respectively [23]. Deviations from Hardy-Weinberg equilibrium (HWE) and linkage disequilibrium (LD) were tested using the online version of GENEPOP [24]. All results were adjusted for multiple simultaneous comparisons using a sequential Bonferroni correction [25]. Genotyping errors due to null alleles, stutter bands, or allele dropout were analyzed using the software Micro-checker 2.2.3 [26].

Cross-species amplification of the above-developed polymorphic SSR loci was tested in four species of sinipercine fishes: *S. kneri*, *S. undulata*, *S. obscura*, and *C. whiteheadi*. Two individuals of each species were analyzed. The same PCR conditions were used as described above except that the annealing temperature was re-optimized at each locus (Table 2). Amplification products were visualized in 1.5% agarose gels, and fragments were sized by comparison with a 2 kb DNA Marker (Trans). Primer pairs that amplified fragments with similar sizes to those observed in source species were considered as successful cross-species amplification.

## 4. Conclusions

In summary, a total of 46 polymorphic EST-SSR markers were newly developed. Forty-three of these loci were polymorphic in *S. chuatsi*, and 20 were polymorphic in *S. scherzeri*. We only tested a small subset of the SSR loci identified in our transcriptome, but high levels of polymorphism, and high level of cross-species amplification indicate that the pairs of primers described here may be suitable for assessments of genetic diversity and population structure, the construction of high-density linkage map, conservation and molecular marker-assisted breeding in many species of sinipercine fishes. Our results highlight the value of next generation transcriptome resources for the characterization and development of gene-based SSRs.

## Figures and Tables

**Table 1:** Characterization of 46 polymorphic EST-simple sequence repeats (SSR) markers in *S. chuatsi* and *S. scherzeri*.

Locus	Accession number	Repeat motif	Primer sequence(5′-3′)	Size range (bp)	*Ta* (°C)	*Na*	*H_O_*	*H_E_*	*PIC*	*p*-Value
Sin109	JQ804765	(AG)_15_	F: GGACACTGGACACTCAAACAT	220–270	54.5	4	0.2500	0.6339	0.5747	1.0000
			R: AGAGGATCAAAATTGTGCTTGAA	246–285	54.5	6	0.6875	0.8170	0.7756	0.9510
Sin110	JQ804766	(AC)_15_	F: TGCTGTTTCCTCAAAACCCCT	177–244	54.5	6	0.7188	0.8194	0.7798	0.9933
			R: AATCCAAGTGACAGGACGCC	—	—	—	—	—	—	—
Sin112	JQ804768	(AC)_15_	F: ATCGGCACCTGAGGCAAAAG	132–166	54.5	6	0.9688	0.7897	0.7444	0.0030
			R: GCCATCCATAGAGCCACGTC	129–198	54.5	9	1.0000	0.8770	0.8479	0.0090
Sin113	JQ804769	(TG)_15_	F: TCCCCATATCTGCCCTGACC	90–126	54.5	6	0.8125	0.7907	0.7439	0.9514
			R: GTGCACATGTCGAGTCAGTA	—	—	—	—	—	—	—
Sin114	JQ804770	(AC)_14_	F: AAGAGACAAGACACCACCGC	185–209	54.5	5	0.4375	0.6736	0.6012	0.9347
			R: ATGGTTTGACGGGAGACAGC	194–243	54.5	7	1.0000	0.8418	0.8064	0.0039
Sin116	JQ804771	(TG)_14_(AG)_7_	F: ACAATCCCAGCCCTCCTTCT	212–265	54.5	6	0.5625	0.8219	0.7813	0.9999
			R: GCAAGGTCCCTTTACATGCAG	219–259	54.5	5	0.8750	0.7877	0.7393	0.0798
Sin117	JQ804772	(GT)_14_	F: GGGCGGAAGACCAACTATGT	268–291	54.5	3	0.4062	0.5332	0.4697	0.9871
			R: TTTCTGTCCTTTTTCCTCTCGC	—	—	—	—	—	—	—
Sin118	JQ804773	(GT)_14_	F: AGGCCACACTTTAGTCACATC	163–192	54.5	4	0.8750	0.7376	0.6754	0.0393
			R: ACCACACTCCAGCATTTCCC	157–189	54.5	3	1.0000	0.6225	0.5378	0.0000 *
Sin119	JQ804774	(CA)_14_	F: AACAACTTTTTACCGCCAGCC	180–226	54.5	4	0.8438	0.7282	0.6652	0.1123
			R: ACCTCTGCTGCACAGCTAATC	—	—	—	—	—	—	—
Sin120	JQ804775	(TTTG)_7_	F: CCATCCCTCCGACCTTCAGT	119–134	54.5	4	0.5312	0.6900	0.6209	0.9418
			R: TTTAGGAACCCGACTCCGCT	—	—	—	—	—	—	—
Sin122	JQ804777	(TG)_14_TAG(GT)_7_	F: TGCACTCACACACCTGTCTC	—	—	—	—	—	—	—
			R: AGCAGGATGCTTCATGCACTT	205–246	54.5	5	1.0000	0.6667	0.5927	0.0000 *
Sin123	JQ804778	(AC)_14_	F: GATGGTGGTGAAACACTGGCT	249–307	54.5	6	0.7812	0.7773	0.7352	0.7478
			R: GTGTTGAGAGGGTCCTGGTG	198–214	56.0	3	0.5000	0.5397	0.4683	0.9912
Sin124	JQ804779	(CA)_14_	F: TCAAACACCACCCACCCCTG	248–281	54.5	4	0.8750	0.7297	0.6667	0.0180
			R: ACCGGGACAGGATGGGAGTC	—	—	—	—	—	—	—
Sin125	JQ804780	(CA)_14_	F: ACCCTCTGTGTGGCGAATGT	277–311	54.5	3	0.6250	0.6265	0.5474	0.5368
			R: CGGGACAGGATGGGAGTCG	—	—	—	—	—	—	—
Sin127	JQ804782	(TG)_14_	F: AGACGTAGCCCAGGCTCAAA	215–251	54.5	3	0.5938	0.5055	0.4213	0.0303
			R: TGTGGGGTTCACTACAGGGT	—	—	—	—	—	—	—
Sin128	JQ804783	(AC)_14_	F: CTGTGCCTCAGTGTGCTGC	225–257	54.5	3	0.4062	0.6394	0.5572	0.9995
			R: ACTTGTAATGGGCAAATTGTCACT	—	—	—	—	—	—	—
Sin129	JQ804784	(CA)_14_	F: ACGCTGCGAGGTGTGATATG	131–164	54.5	5	0.6250	0.7500	0.6971	0.9857
			R: CTGGCCCTCGTTAGTGCTTG	185–217	54.5	8	1.0000	0.8457	0.8103	0.0046
Sin130	JQ804785	(GTGA)_7_N_7_(TG)_8_	F: CTCGCAGGCTTTTCTCTGCT	282–300	54.5	2	0.3438	0.3963	0.3140	0.8902
			R: AGCCATCAGTTCTGTTCTTTCTT	252–280	54.5	5	0.7188	0.7485	0.6982	0.3062
Sin131	JQ804785	(ATGG)_7_	F: GGAGGAAAATAATTTCATTTGGGAT	180–200	54.5	3	0.1250	0.4107	0.3665	0.9998
			R: GTCATTGCATTCAAAAGTTAGGCT	—	—	—	—	—	—	—
Sin134	JQ804789	(TG)_14_	F: GCCCCCTTCTCAACCCACTA	106–120	54.5	6	1.0000	0.8075	0.7645	0.0008 *
			R: TGCTTTCCAAAGCGAACCGT	108–134	54.5	8	0.9688	0.8621	0.8299	0.0451
Sin135	JQ804790	(TG)_14_	F: GTGATATCTCCTCCTGACGGC	273–302	54.5	4	0.5938	0.5585	0.4900	0.3484
			R: ACATTCTGAATTGCAAAGGCTCA	—	—	—	—	—	—	—
Sin136	JQ804791	(TG)_14_	F: AACTGAAATGTGTGGTGAACTGA	138–164	56.8	5	0.5000	0.7202	0.6585	0.9243
			R: GTGTCTCCCAACAAGTGGCA	—	—	—	—	—	—	—
Sin137	JQ804792	(TCA)_9_	F: AGCGTCTACTGAGGGTCAAACT	234–280	54.5	5	0.4688	0.7207	0.6700	0.9960
			R: GGTGGACTGACCAGCAAGGA	—	—	—	—	—	—	—
Sin138	JQ804793	(ATC)_9_	F: TCATCTGAGGACGACTCGCT	229–253	52.5	3	0.4375	0.5997	0.5025	0.9772
			R: AACTTAACTTCCTGCTGTCCCT	—	—	—	—	—	—	—
Sin139	JQ804794	(CTC)_9_	F: GTGACTGCATCCAGGTGTCG	—	—	—	—	—	—	—
			R: GGCCGAGGTCGGTTGTTATC	189–207	54.5	3	0.1875	0.2803	0.2584	0.9947
Sin140	JQ804795	(TCA)_9_	F: TGTGGTTCTCCTCTCCCACA	253–304	53.2	5	0.4375	0.7336	0.6759	0.9985
			R: AGAGGTTGGTGCAGGAGACTT	—	—	—	—	—	—	—
Sin142	JQ804797	(CTT)_9_	F: CATCAACGCAATGCAAGGGT	150–180	54.5	2	0.1562	0.3289	0.2713	0.9997
			R: CTGGAGCCGGACTTGAGGAA	181–226	54.5	6	0.3438	0.6994	0.6492	0.9998
Sin143	JQ804797	(GTT)_7_	F: AAAGCAGGCCAAACAACACC	198–246	54.5	5	0.4062	0.7733	0.7206	1.0000
			R: AGGACGGGGAGGCTTTTGAT	—	—	—	—	—	—	—
Sin146	JQ804800	(GAG)_6_ N_5_ (AAG)_9_	F: GTAATCGACACGGACAGCGA	370–452	54.5	4	0.5938	0.6781	0.6069	0.8240
			R: CACACACATTCTCCTCAGCGT	—	—	—	—	—	—	—
Sin147	JQ804801	(TCC)_9_	F: AGATCAGACACCAGGAGGACC	174–232	53.5	5	0.3125	0.7242	0.6576	0.9971
			R: AAGACGGAGGCAAAGAACGAC	192–225	54.5	4	0.5625	0.7614	0.7026	0.9964
Sin148	JQ804802	(CAT)_9_	F: CGAGGCCAGGAGTGAACCAA	255–303	53.5	3	0.6875	0.4866	0.4009	0.0027
			R: GCACAGCTGGAGGTGTTTCG	—	—	—	—	—	—	—
Sin151	JQ804805	(GT)_13_	F: GTGCAAGGCCTTAGTCTCTCC	170–221	55.5	4	0.6875	0.6434	0.5761	0.8897
			R: GCCCACCAGATCTACCGAGT	—	—	—	—	—	—	—
Sin152	JQ804806	(AG)_8_A(AG)_13_	F: TGCGCCACTTTACTGATGGG	173–242	53.5	6	0.6562	0.8105	0.7677	0.9164
			R: GCATTAACCAAACCCCGCGA	185–240	54.5	8	0.9688	0.8522	0.8193	0.1740
Sin153	JQ804807	(AG)_13_	F: GCACAGGTTTTTCTAAACATTGCT	155–208	53.5	5	0.1875	0.3795	0.3538	0.9662
			R: TGTTGTTATTGTCAGTGTGTTTTCT	177–215	54.5	4	0.2500	0.6806	0.6160	1.0000
Sin154	JQ804808	(GT)_13_	F: ACTGGTTTGTGGTTTGGAGGT	211–229	53.5	2	0.5625	0.4107	0.3225	0.0356
			R: ATGATTTTTCTTGCCTTCGTGT	—	—	—	—	—	—	—
Sin155	JQ804809	(AC)_13_	F: GAATGGTGTGTTGCACAGCG	157–190	53.5	3	0.2188	0.3894	0.3473	0.9939
			R: CATTCTAGCATGTGCGAGGC	160–201	54.5	7	0.6875	0.8021	0.7600	0.9004
Sin156	JQ804810	(AC)_13_	F: TAGGAGGCTTTACAACCGGC	188–205	53.5	2	0.5625	0.4107	0.3225	0.0344
			R: ATGACCAGCCTCAGGTGTCT	—	—	—	—	—	—	—
Sin157	JQ804811	(AC)_13_	F: CATTTGCTGGCTCTCACACC	184–215	53.5	3	0.5000	0.4330	0.3477	0.2070
			R: TGTTTAATTCATGCCTAGGTTTAGT	—	—	—	—	—	—	—
Sin158	JQ804812	(CA)_13_	F: TGAGAACTGCCTGAGCCGAG	—	—	—	—	—	—	—
			R: CTGCAGAGCCGTGGAGACTA	210–248	54.5	3	0.9062	0.5303	0.4145	0.0000 *
Sin159	JQ804813	(TG)_13_	F: CGCTGATCGCTCTGTGCTCCC	196–234	53.5	5	0.6875	0.7614	0.7108	0.7259
			R: ACACGGAAGCTGGTGAGCGG	199–233	56.0	5	0.9688	0.6379	0.5682	0.0000 *
Sin160	JQ804814	(TG)_13_	F: CCACTGGAGCCCACATGGCA	307–360	55.5	5	0.8125	0.6711	0.6123	0.0151
			R: TGAGTGGGCGCTACTGTGTGT	291–331	54.5	5	0.7812	0.7659	0.7137	0.5928
Sin162	JQ804815	(TA)_13_	F: TGCTTTGCTGGTTGGCAGGCT	294–368	53.5	5	0.1875	0.6270	0.5651	1.0000
			R: CGTGGAGGTGCGACGCGTAA	—	—	—	—	—	—	—
Sin163	JQ804817	(CA)_13_	F: ACAGCCAGGCTCCTCCACCT	230–269	53.5	8	0.6875	0.8482	0.8134	0.9597
			R: TCTTTCACAGGCAAACCACTGCT	225–273	53.5	6	0.4688	0.7803	0.7392	1.0000
Sin166	JQ804819	(GA)_13_	F: GAAATTGAAGAAGACAAGGTGATG	204–231	53.5	3	0.2500	0.4504	0.4012	0.9998
			R: CTGCTTTTGGCAGGAGCTAA	—	—	—	—	—	—	—
Sin169	JQ804822	(AC)_13_	F: TGACAAATCACTGGGTTTACTCCT	214–284	53.5	5	0.5625	0.6443	0.5790	0.9164
			R: GACATGCTGCTCTCCGATCC	—	—	—	—	—	—	—
Sin170	JQ804823	(GT)_13_	F: CTTGAGTGGTTGATTGTGCCCT	242–270	55.5	4	0.7188	0.5491	0.4990	0.0015
			R: GCAGACATTGCTGAGGGATGAA	—	—	—	—	—	—	—

For each locus the information in the top row refers to *S. chuatsi* and the second row refers to *S. scherzeri. Ta* corresponds to annealing temperature; *Na* is number of alleles; *H_O_* and *H_E_* are observed and expected heterozygosity, respectively; *PIC* is the polymorphic information content.

* indicates significant deviation from HWE after Bonferroni correction; no polymorphism for each locus is denoted by “—”.

**Table 2:** Cross-species amplification for the 46 polymorphic EST-SSR markers in four species of sinipercine fishes.

	Species			
	
Locus	*S. undulate*	*S. obscura*	*S. kneri*	*C. whiteheadi*
Sin109	54.5	54.5	54.5	54.5
Sin110	54.5	54.5	54.5	54.5
Sin112	54.5	54.5	54.5	54.5
Sin113	54.5	54.5	54.5	54.5
Sin114	54.5	54.5	54.5	54.5
Sin116	54.5	54.5	54.5	—
Sin117	54.5	54.5	54.5	—
Sin118	54.5	54.5	54.5	54.5
Sin119	54.5	54.5	54.5	54.5
Sin120	54.5	54.5	54.5	54.5
Sin122	54.5	54.5	54.5	54.5
Sin123	54.5	54.5	54.5	54.5
Sin124	54.5	54.5	54.5	54.5
Sin125	54.5	54.5	54.5	54.5
Sin127	54.5	54.5	54.5	54.5
Sin128	54.5	54.5	54.5	—
Sin129	54.5	54.5	—	54.5
Sin130	54.5	54.5	54.5	54.5
Sin131	54.5	54.5	54.5	54.5
Sin134	54.5	54.5	54.5	54.5
Sin135	54.5	54.5	54.5	—
Sin136	54.5	54.5	54.5	54.5
Sin137	54.5	54.5	54.5	54.5
Sin138	54.5	54.5	54.5	54.5
Sin139	54.5	54.5	54.5	54.5
Sin140	54.5	54.5	54.5	54.5
Sin142	54.5	54.5	54.5	54.5
Sin143	54.5	54.5	54.5	54.5
Sin146	54.5	54.5	54.5	—
Sin147	54.5	54.5	54.5	54.5
Sin148	54.5	54.5	54.5	54.5
Sin151	54.5	54.5	54.5	54.5
Sin152	54.5	54.5	54.5	54.5
Sin153	54.5	54.5	54.5	54.5
Sin154	54.5	54.5	54.5	54.5
Sin155	—	—	—	—
Sin156	54.5	54.5	54.5	54.5
Sin157	54.5	54.5	54.5	54.5
Sin158	54.5	54.5	54.5	54.5
Sin159	54.5	54.5	54.5	52.8
Sin160	54.5	54.5	54.5	—
Sin162	54.5	54.5	57.0	51.1
Sin163	54.5	54.5	54.5	52.8
Sin166	54.5	54.5	54.5	54.5
Sin169	54.5	54.5	54.5	54.5
Sin170	54.5	54.5	54.5	54.5

The annealing temperature for each locus was shown. Unsuccessful amplification of PCR products for each locus is denoted by “—”.

## References

[1] Liang Y., Cui X. (1982). The eco-physiological characteristics of artificial propagation in mandarin fish (*Siniperca chuatsi*). Acta Hydrobiol. Sin.

[2] Liu J., Cui Y., Liu J. (1998). Food consumption and growth of two piscivorous fishes, the mandarin fish and the Chinese snakehead. J. Fish Biol.

[3] He J.G., Zeng K., Weng S.P., Chan S.M. (2002). Experimental transmission, pathogenicity and physical-chemical properties of infectious spleen and kidney necrosis virus (ISKNV). Aquaculture.

[4] Zhang C., Zhao Y. (1999). The resources status, recovery and reasonable utilization of *Siniperca chuatsi* in China (in Chinese). Bull. Biol.

[5] Walter R., Epperson B.K. (2001). Geographic pattern of genetic variation in *Pinus resinosa*: Area of greatest diversity is not the origin of postglacial populations. Mol. Ecol.

[6] Saha M.C., Cooper J.D., Rouf Mian M.A., Chekhovskiy K., May G.D. (2006). Tall fescue genomic SSR markers: Development and transferability across multiple grass species. Theor. Appl. Genet.

[7] Edwards K.J., Barker J.H., Daly A., Jones C., Karp A. (1996). Microsatellite libraries enriched for several microsatellite sequences in plants. BioTechniques.

[8] Gupta P.K., Rustgi S. (2004). Molecular markers from the transcribed/expressed region of the genome in higher plants. Funct. Integr. Genomics.

[9] Saha M.C., Mian M.A., Eujay I., Zwonitzer J.C., Wang L., May G.D. (2004). Tall fescue EST-SSR markers with transfer ability across several grass species. Theor. Appl. Genet.

[10] Zhang B., Li Z.J., Tong J.G., Liao X.L. (2006). Isolation and characterization of 18 polymorphic microsatellite markers in Chinese mandarin fish *Siniperca chuatsi* (Basilewsky). Mol. Ecol. Notes.

[11] Kuang G.Q., Liu Z., Lu S.Q., Liu H.Y., Zhang J.S., Tang J.Z. (2007). Isolation and characterization of microsatellite loci from *Siniperca chuatsi* (in Chinese). J. Fish Sci. China.

[12] Kuang G.Q., Liu Z., Lu S.Q., Liu H.Y., Zhang J.S., Xiao T.Y. (2007). Isolation and characterization of microsatellite loci of *Siniperca chuatsi* from GenBank database (in Chinese). Acta Zool. Sin.

[13] Kuang G.Q., Lu S.Q., Zheng S.M., Wu Q. (2009). Isolation and evaluation of 18 microsatellite loci in *Siniperca chuatsi* (Basilewsky). Mol. Ecol. Resour.

[14] Liu X.L., Luo W., Zeng C., Wang W.M., Gao Z.X. (2011). Isolation of New 40 Microsatellite Markers in Mandarin Fish (*Siniperca chuatsi*). Int. J. Mol. Sci.

[15] Yang M., Liang X.F., Tian C.X., Gul Y., Dou Y.Q., Cao L., Yu R (2012). Isolation and characterization of fifteen novel microsatellite loci in golden mandarin fish (*Siniperca scherzeri*) Steindachne. Conserv. Genet. Resour.

[16] Liu H.Z. (1993). Studies on Skeleton Anatomy and Phylogeny of the Sinipericine Fishes. Ph.D. Dissertation.

[17] Barbara T., Palma-Silva C., Paggi G.M., Bered F., Fay M.F., Lexer C. (2007). Cross-species transfer of nuclear microsatellite markers: potential and limitations. Mol. Ecol.

[18] Ellis J.R., Burke J.M. (2007). EST-SSRs as a resource for population genetic analyses. Heredity.

[19] Wang X.W., Luan J.B., Li J.M., Bao Y.Y., Zhang C.X., Liu S.S. (2010). *De novo* characterization of a whitefly transcriptome and analysis of its gene expression during development. BMC Genomics.

[20] You F.M., Huo N., Gu Y.Q., Luo M.C., Ma Y., Hane D., Lazo G.R., Dvorak J., Anderson O.D. (2008). BatchPrimer3: A high throughput web application for PCR and sequencing primer design. BMC Bioinforma.

[21] National Center for Biotechnology Information, U.S. National Library of Medicine http://www.ncbi.nlm.nih.gov/tools/primer-blast/index.cgi?LINK_LOC=BlastHome.

[22] Yeh F.C., Boyle T.J.B. (1997). Population genetic analysis of co-dominant and dominant markers and quantitative traits. Belg. J. Bot.

[23] Botstein D., White R.L., Skolnick M., Davis R.W. (1980). Construction of a genetic linkage map in man using restriction fragment length polymorphisms. Am. J. Hum. Genet.

[24] Raymond M., Rousset F. (1995). GENEPOP (version 1.2): Population genetic software for exact tests and ecumenicism. J. Hered.

[25] Rice W.R. (1989). Analyzing tables of statistical tests. Evolution.

[26] Van Oosterhout C., Hutchinson W.F., Wills D.P.M., Shipley P. (2004). MICRO-CHECKER: Software for identifying and correcting genotyping errors in microsatellite data. Mol. Ecol. Notes.

